# Supplementary material for: Anatomy-aware and acquisition-agnostic joint registration with SynthMorph
Source: Imaging Neurosci (Camb). 2024 Jun 25;2:imag-2-00197. doi: 10.1162/imag_a_00197 (PMC11247402; doi:10.1162/imag_a_00197)
Supplement: Supplementary Material [file imag_a_00197-supp.pdf]

Supplementary material

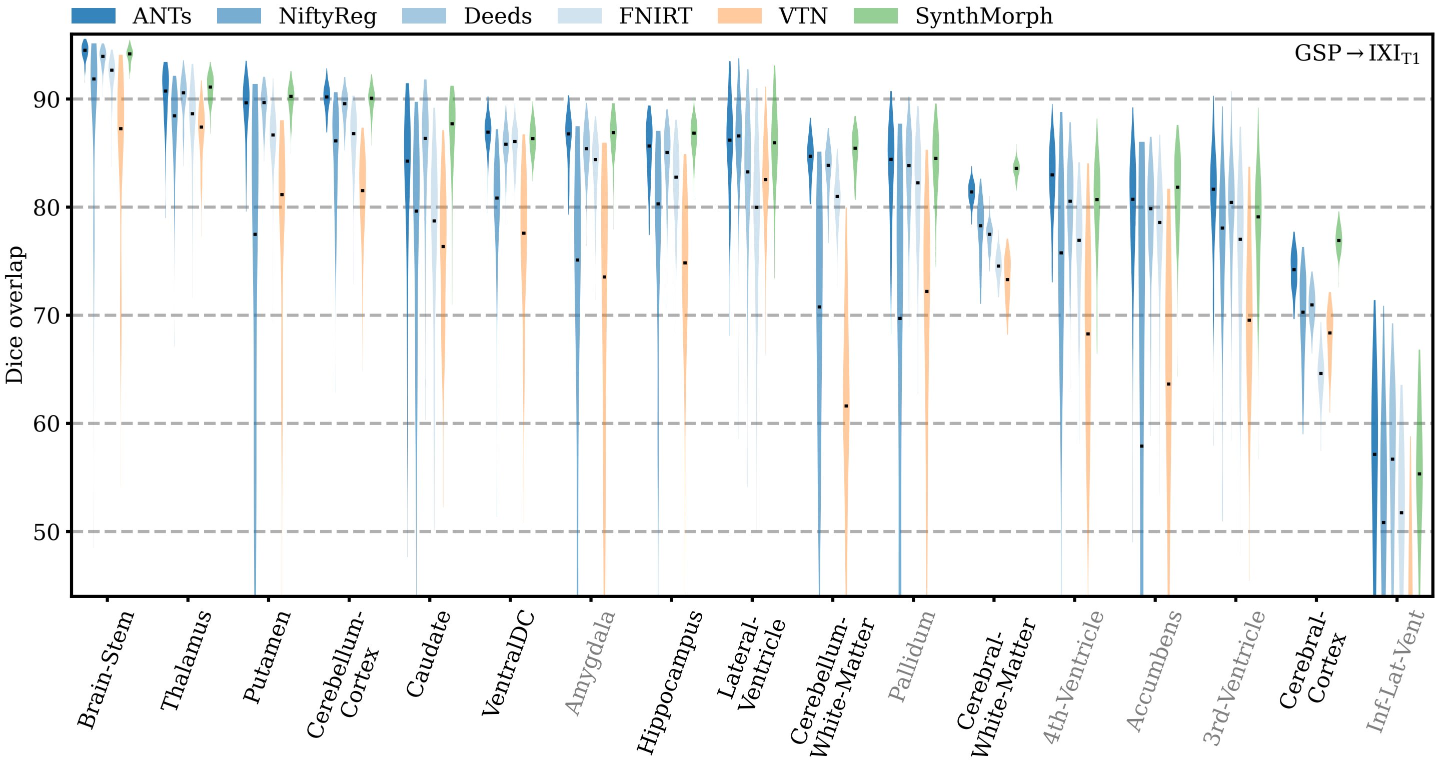

Figure S1. Deformable 3D registration accuracy per structure across testset GSP→IXI$_{T1}$ after affine initialization with NiftyReg. We left-right average bilateral brain regions. SynthMorph training did not optimize the overlap of structures shown in gray.

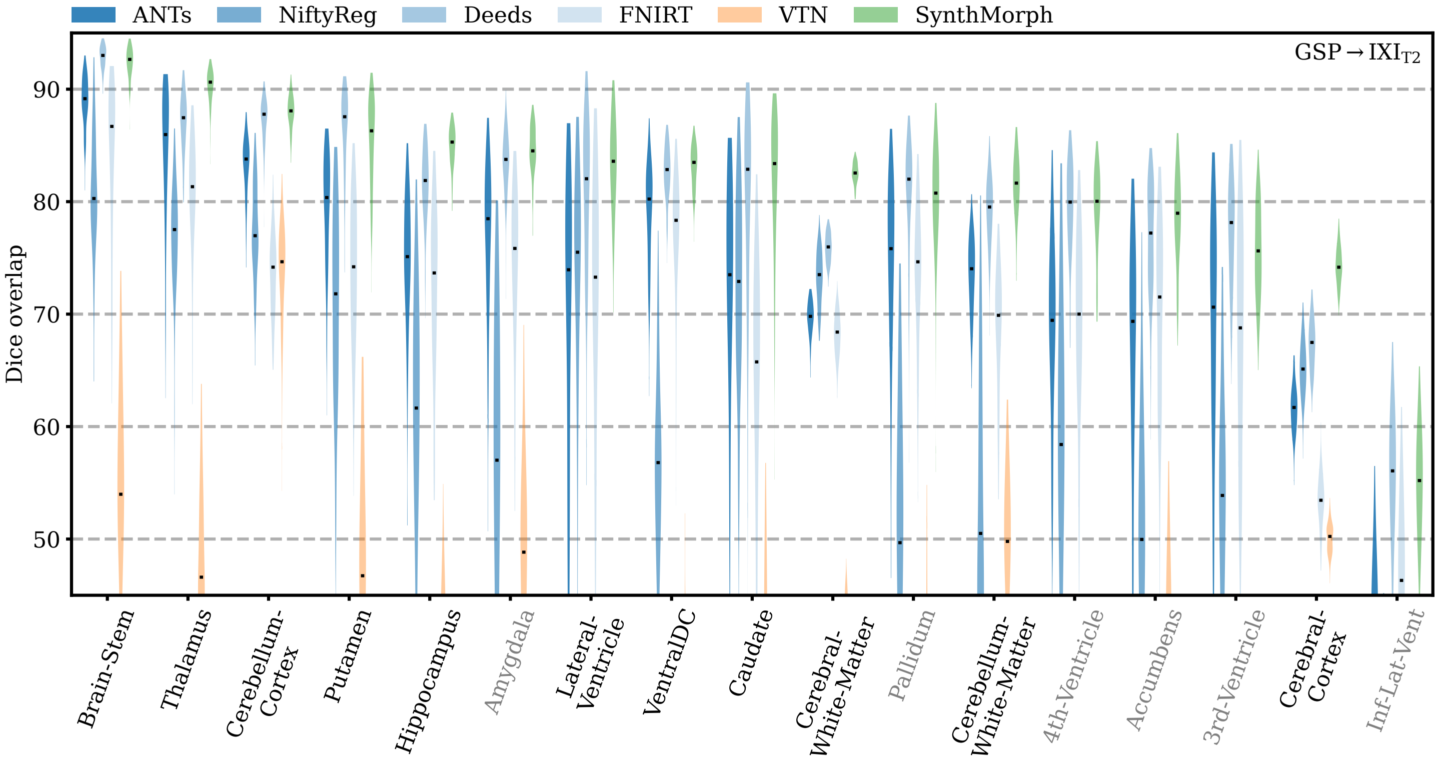

Figure S2. Deformable 3D registration accuracy per structure across testset GSP→IXI$_{T2}$ after affine initialization with NiftyReg. We left-right average bilateral brain regions. SynthMorph training did not optimize the overlap of structures shown in gray.

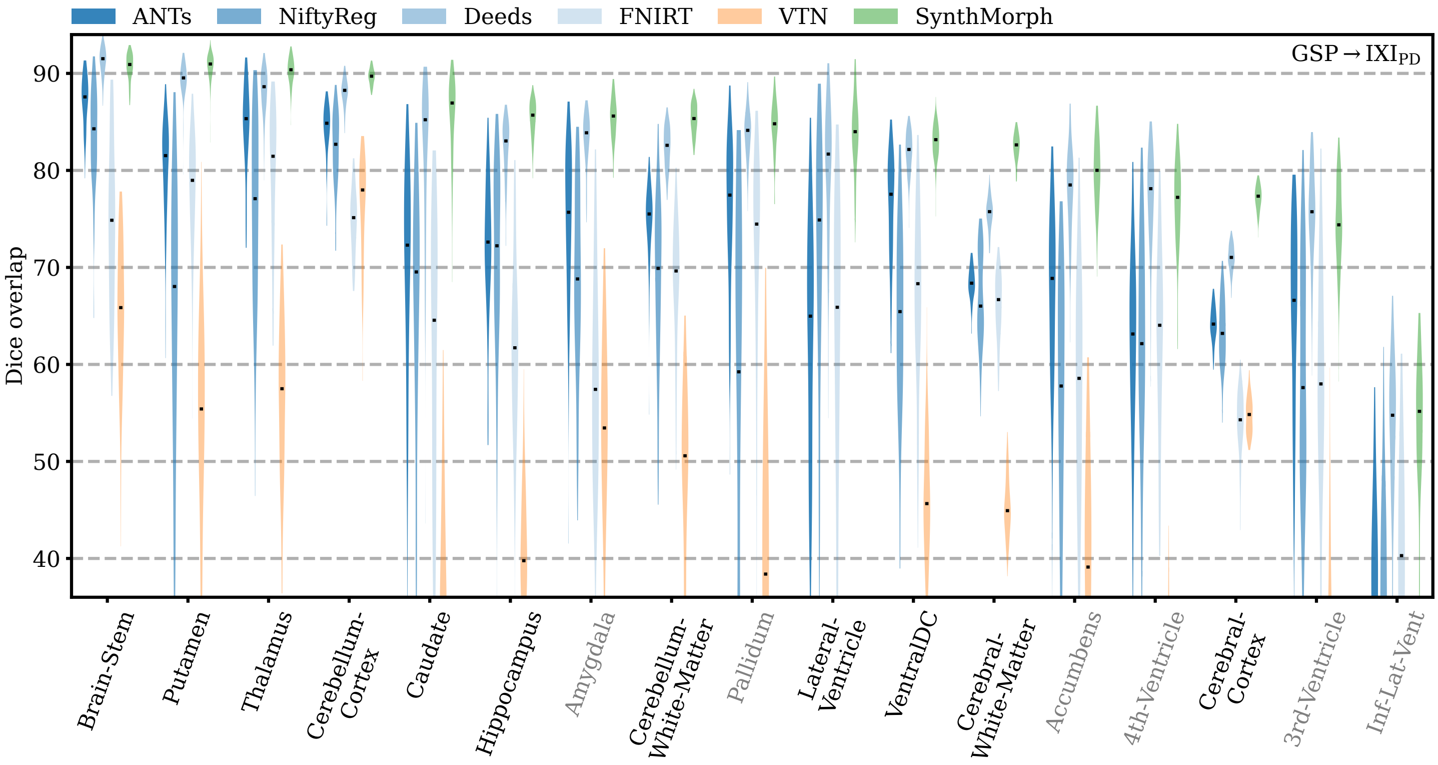

Figure S3. Deformable 3D registration accuracy per structure across testset GSP→IXI$_{PD}$ after affine initialization with NiftyReg. We left-right average bilateral brain regions. SynthMorph training did not optimize the overlap of structures shown in gray.

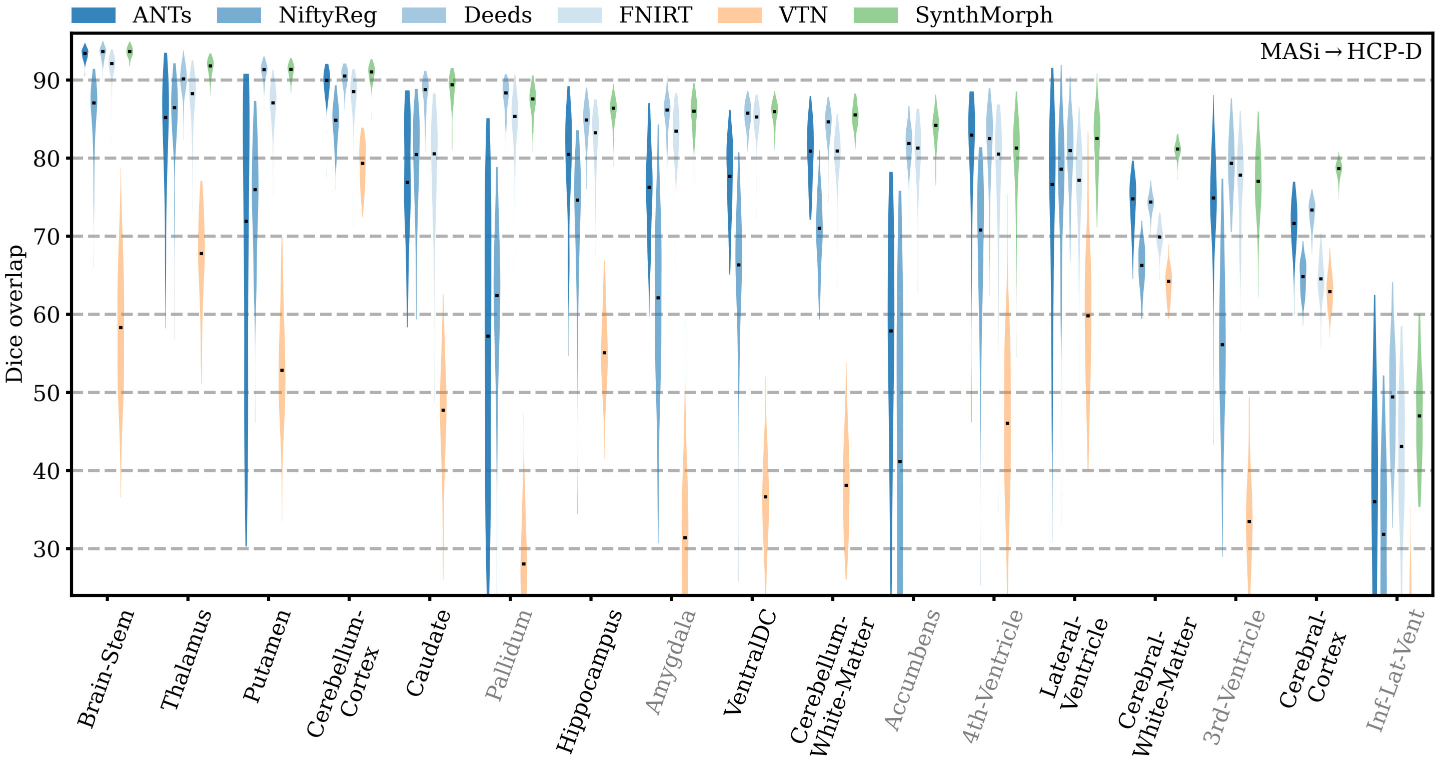

Figure S4. Deformable 3D registration accuracy per structure across testset MASi→HCP-D after affine initialization with NiftyReg. We left-right average bilateral brain regions. SynthMorph training did not optimize the overlap of structures shown in gray.

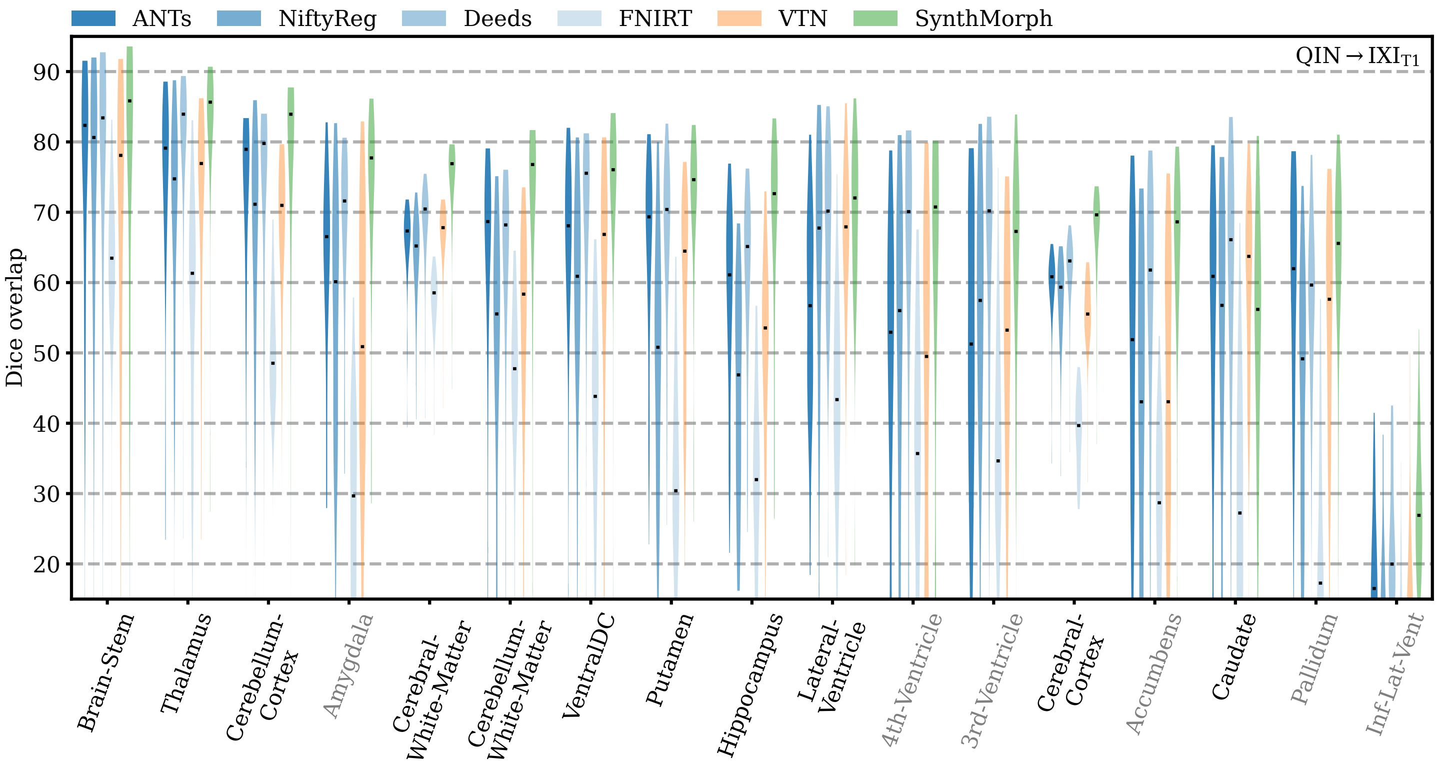

Figure S5. Deformable 3D registration accuracy per structure across testset QIN→IXI$_{T1}$ after affine initialization with NiftyReg. We left-right average bilateral brain regions. SynthMorph training did not optimize the overlap of structures shown in gray.
